# Development and assessment of a Mentor Training Workshop Series and Certificate Program

**DOI:** 10.1017/cts.2025.71

**Published:** 2025-04-16

**Authors:** Kenzie A. Cameron, Mercedes R. Carnethon, Morgan Barrowman, Leah J. Welty

**Affiliations:** 1 Division of General Internal Medicine, Department of Medicine, Northwestern University Feinberg School of Medicine, Chicago, IL, USA; 2 Department of Preventive Medicine, Northwestern University Feinberg School of Medicine, Chicago, IL, USA; 3 Northwestern University Clinical and Translational Sciences Institute, Chicago, IL, USA

**Keywords:** Mentoring, mentor training, faculty development, CTSA, assessment

## Abstract

Within the Northwestern University Clinical and Translational Sciences Institute, we created a foundational and fluid mentor training curriculum to provide competency-based mentor training for faculty. Via our “Developing and Enhancing Mentoring Relationships” mentor workshop series, launched in 2020, we present eight 90-minute workshops each academic year. This series is designed for both training naive participants and previously trained mentors across ranks and tracks and offers both repeating (“required”) and new (“elective”) workshops annually. We implemented a Mentor Training Certificate Program (MTCP) in 2021 to formally recognize faculty who complete a minimum of nine hours of training, consisting of three required and at least three elective training sessions. Over the first four years of the workshop series, 345 unique faculty attended at least one workshop; 46 completed requirements for the MTCP. MTCP participants complete baseline and annual surveys focused on self-assessment of nine mentoring skills (e.g., providing feedback) and frequency of engaging in five recommended mentoring behaviors (e.g., aligning expectations). Scores increased significantly across all skills; participants reported increased frequency of mentoring behaviors and high satisfaction with the program. Our mentor workshop series, offering both repeating and new workshops annually, provides faculty the opportunity for either initial or advanced training.

## Introduction

The value of effective mentoring for the retention and success of early career academic faculty has prompted institutions and consortiums to think strategically about how to train and support faculty mentors [1–6]. The proliferation of such programming pivots away from believing that some faculty are naturally “good” at mentoring. More institutions are investing in structured mentor training programs and curricula such as *Entering Mentoring* [7], originally created for the context of the biological sciences, and focusing on the science of mentorship [8].

Recognizing the need for training across academic disciplines, a multi-institutional team led by scholars at the University of Wisconsin-Madison created *Mentor Training for Clinical and Translational Researchers,* an 8-hour case-based training curriculum [1,2]. This seminal program, targeting clinical and translational researchers, was tested in a randomized clinical trial (RCT) at 16 academic institutions [2,6]. Given its success in improving mentors’ skills via competency-based training, the curriculum has been widely disseminated and adapted to local institutional contexts. One common adaptation is institutional recognition that educators and clinicians also contribute to the research capacity and need mentor training. Federal funders of academic training programs increasingly expect that program directors and mentors demonstrate that they have undergone mentor training [9]. Consequently, there is a need to provide training in mentoring best practices for faculty and to certify receipt of training.

Our large academic medical center participated in the mentor training RCT in 2010–2011 [2,6]. Given participants’ positive feedback, and an institutional commitment to improving mentoring, we launched a revised and abbreviated 4-hour version. Between 2013 and 2015, this training was provided to faculty across ranks who hold appointments in the Northwestern University Feinberg School of Medicine (NUFSM). From 2015 to 2019, we provided annual workshops for new faculty and those wishing a refresher of skills. Over time, attendance dwindled to approximately 30 faculty per year. Based on participant feedback, we identified a need for mentor training across ranks and tracks, coupled with a desire for mentoring competencies to be explored across different contexts (e.g., research, clinical, and education). Previous attendees were interested in returning for training but wanted content beyond that previously presented and options beyond an annual half-day session.

In response, our team of investigators in the Northwestern University Clinical and Translational Science (NUCATS) Institute created the “Developing and Enhancing Mentoring Relationships” mentor workshop series. To meet local institutional needs, we provided mentor training for both training naive participants and previously trained mentors and developed a certificate program to formally recognize faculty who completed training. Our objectives in this manuscript are to 1) describe the structure and content of the mentoring series and certificate program; 2) describe program participants; and 3) present our assessment following the first four years of the program.

## Methods

### Design of the Developing and Enhancing Mentoring Relationships mentor workshop series/Mentor Training Certificate Program (MTCP)

Our goal in creating the mentor workshop series, with an option to enroll in the Mentor Training Certificate Program (MTCP), was to provide competency-based ongoing mentor training for faculty across ranks and faculty tracks (e.g., investigator, clinician educator, team science, and research track). We sought to build a foundational and fluid curriculum, including specific core “required” sessions (repeated annually), and “elective” sessions, designed to vary year to year. Required sessions include *Establishing and Aligning Expectations in Mentoring Relationships*, *Cultural Awareness in Mentoring Across Differences*, and *Articulating Your Mentoring Philosophy and Plan.* These sessions were designed, in part, based on the successful *Mentor Training for Clinical and Translational Researchers* curriculum [2,6]. Additional workshops explored goal setting and evaluation, using coaching skills in mentoring relationships, effective communication, difficult conversations and conflict, fostering independence, giving meaningful feedback, and mentor networks and mentor mapping, among other topics (see Table 1).

**Table 1.** Mentoring series workshop topics, format, and number of attendees by year (2020–2024)^1,2^

Topic	2020 – 2021	2021 – 2022	2022 – 2023	2023 – 2024
Articulating your Mentoring Needs	32 (V)			
**Establishing and Aligning Expectations in Mentoring Relationships**	31 (V)	27 (V)	16 (IP)	62 (V)
Maintaining Effective Communication/Difficult Conversations	25 (V)	26 (V)		
**Cultural Awareness to Enhance Mentoring Across Differences**	26 (V)	24 (V) 8 (V)	15 (IP)	39 (IP)
Creating SMART Goals	20 (V)			
Mentor Mapping and Developmental Networks	26 (V)			
Evaluating your Goals/Evaluating your Mentee’s Goals	8 (V)			
**Articulating your Mentoring Philosophy and Plan**	21 (V)	17 (V) 8 (IP)	13 (IP)	34 (IP)
Work-Life Integration		23 (V)		
Fostering Independence/Transitioning your Mentee to Independence		24 (V)		
Dealing with Mentoring Challenges		23 (V)		
Goal Planning at Different Stages of One’s Career		28 (V)		
Being and Ally and a Sponsor (Panel)			37 (V)	
Strategic Mentoring: Using Mentoring Families for Increased Benefit			15 (IP)	
Applying Coaching Skills in Mentoring Relationships^3^			26 (V)	
Developing and Evaluating your Mentee’s Goals for Career Progression			28 (V)	
Navigating Conflict in the Mentor–Mentee Relationship			18 (V)	
How Coaching can Help you Become a Better Mentor				27 (IP)
Navigating Turbulent Waters: Conflict Management in Mentoring Relationships^3^				56 (V)
Giving Meaningful Feedback				67 (V)
Mentoring in Mid-Career				25 (IP)
Team-Based Mentoring				24 (IP)

1 Workshops required to complete the Mentor Training Certificate (bolded) are offered annually.

2 (V) indicates offered virtually, (IP) indicates workshop conducted in-person. Our Cultural Awareness session was offered virtually twice in 2021 – 2022 as we were unable to host it in-person due to the pandemic. We were able to offer our Articulating workshop both virtually and in person that year.

3 Session was presented by colleagues external to NUFSM.

The workshop series launched in September 2020, offering eight monthly workshops through June 2021. This series consists of 90-minute workshops designed to prepare mentors and mentees to be effective and successful in their roles. Attendees were welcome to attend as many workshops as fit their needs. The structure of the workshops generally followed a format of (1) didactic presentation of evidence-based best practices, (2) small group activities focused on relevant case studies or discussion prompts, (3) large group debrief, and (4) introduction to tools (e.g., templates, links to additional resources, exemplary documents, etc.) to assist attendees in implementing best practices (see supplementary appendix for sample agenda and case study).

We implemented the MTCP in 2021 to recognize attendees’ continued involvement in the program and provide confirmation of their participation. To earn the certificate, faculty must (1) participate in the three required workshops (4.5 hours); (2) participate in at least three elective workshops (4.5 hours, selected from any other workshops offered); and (3) complete a baseline and annual survey and evaluations of each workshop attended. Faculty have three years to complete the certificate.

As the launch of the Mentoring Series was during the COVID pandemic, the first two years of the program were conducted virtually via the Zoom platform. During the 2022–2023 academic years, workshops were *either* held in person or virtually.

### Workshop attendees

Mentoring workshops were open to faculty of all ranks and career tracks who hold NUFSM appointments, are members of the Northwestern University CTSA, or who collaborate with faculty involved in biomedical research. Our intended audience was mentors of early career faculty, and we focused our case studies and applications within the context of research mentoring. However, given the applicability of mentoring skills and competencies across contexts, all NUFSM faculty were eligible to participate, regardless of track or career focus (e.g., research, clinical, and education). We limited attendees to faculty, as we wanted to provide a safe space for faculty to acknowledge they may never have received any training and to discuss mentoring challenges they face.

### Mentoring workshop series assessment

To fulfill MTCP requirements, participants were required to complete baseline and annual web surveys (via REDCap [10]) and provide feedback on each workshop attended. Those completing requirements over multiple academic years were asked to complete an annual evaluation.

At baseline, participants completed demographic measures, self-assessments of their mentoring skills, and reported their frequency of engaging in specific mentoring behaviors. These measures were repeated in the annual survey, which also asked participants to indicate their likelihood to recommend the mentoring series, and perception of value of the series. Open-ended items asked participants to identify “mentoring pearls” gained from their involvement and to provide feedback, about the series, as well as suggestions for future workshop topics.

Our survey included relevant items drawn from the Mentorship Competency Assessment [11] that best aligned with planned workshop content. We used three items verbatim *(employing strategies to improve communication, coordinating effectively with other mentors, helping mentees balance work and personal life),* and slightly modified two items *(providing constructive feedback (positive or negative), considering conscious and unconscious biases brought to mentoring relationships).* Given the focus of our workshops, we added an item focused on *engaging mentees in articulating their mentoring needs.* Upon review of our assessment after two years, we created three additional items tailored to mentoring skills highlighted in our required sessions: *building rapport with mentees, distinguishing between a mentoring philosophy and plan,* and *intentionally creating opportunities for mentees to bring up issues of race/ethnicity and other social identities* (see supplementary appendix). Although the original assessment in the MCA provided a 7-point scale, for ease of administration, we asked mentors to use a five-point Likert-type scale (1 = not at all skilled, 3 = moderately skilled, 5 = extremely skilled).

Other items relating to *aligning expectations, discussing mentee(s)’ path to professional independence, setting SMART goals,* and *identifying strategies to reach goals* were adapted from the MCA. However, we asked participants to reflect on *frequency of a behavior* (Never, Once in the beginning, Annually, Quarterly, Monthly, Other, Not applicable), as opposed to rating their perceived skill. We added an item assessing frequency of *evaluating progress toward identified goals.* Additional items queried the frequency of specific behaviors related to the use of Individual Development/Career Plans (IDPs) and meeting agendas; mentors were asked to respond: Never, Seldom, About half the time, Usually, Always, Not applicable. The Northwestern University Institutional Review Board determined the evaluation and assessment of the MTCP to be “Not Human Research.”

### Statistical analyses

We conducted analyses in Stata, version 17 and R, version 4.4.1, with StatTag, version 7 [12–14] to connect statistical output to manuscript text. We summarized categorical variables with frequencies and percentages and Likert scale outcomes with means and standard deviations. We used paired t-tests to examine changes in mentoring skills from baseline to program completion. We used mixed effects ordinal logistic regression with a random intercept for participant to examine within person changes in ordered frequency (e.g., Never, Once, Annually, Quarterly, Monthly) of behaviors with mentees. We also report percentages for frequency of behaviors with mentees. For mentors who took several years to complete the program, we used data from the annual survey they most recently completed.

Our analyses are based on 46 participants who identified as mentors and completed the MTCP. Missing data varied across assessment. All participants completed baseline and annual assessments for most mentoring skills, with the exception of 3 questions which were not initially asked at baseline and therefore available on fewer participants (*building rapport*, *n* = 13; *distinguishing between mentoring plan/philosophy, n* = 12; *creating opportunities, n* = 19). Mentors could respond to questions about frequency of behaviors with mentees as “not applicable” and “other.” In addition, a survey programming error resulted in 5 participants not being asked all items; participants also were not asked these questions if they were not actively mentoring at the time (n = 2). Analyses presented of frequency of behaviors exclude these values as missing and we list sample sizes accordingly.

## Results

Between October 2019 and June 2024, we presented 22 unique workshops. During this time, 345 unique faculty attended at least one session; 289 completed the baseline survey, of whom 124 completed an annual survey. Forty-six mentors completed MTCP, 67 remain active in the program (e.g., completed baseline and annual survey, and attended within the past 2 years). Annual attendance has increased from 99 unique attendees in 2020–2021 to 183 unique attendees in 2023 – 2024. Table 1 notes the format (virtual or in-person) and attendance at each session. Presenters (*n* = 35) have included Department Chairs, Associate and Vice Deans, Directors of multiple NIH Institutional Training Grants, as well as faculty with content expertise (e.g., certified coaches, Center for Leadership faculty). Starting in 2022, one session a year has featured invited colleagues from other institutions and CTSAs.

Table 2 shows demographic and professional characteristics of the 46 participants who completed requirements for the MTCP. Just over half were Assistant Professors, and a quarter were Associate. Fifty percent of participants identified as mentors; the remaining 50% reported being both mentee and mentor (64% of early career faculty, 26% of associate/full professors). The most common career track was Clinician Educator (43%), followed by Research Track (20%) and Investigator/Tenure Track (17%). Half of participants reported 1 – 5 years’ experience mentoring, and another 20% reported >5 – 10 years’ experience; 13% reported no formal experience. Almost all participants (96%) were currently serving as mentors; 44% reported mentoring other faculty, the remainder reported mentoring post-doctoral fellows (research or clinical)/residents, predoctoral students, or staff. Nearly two-thirds of participants identified as women. The majority of participants were White (72%), with relatively smaller attendance by Asian and Hispanic or Latino/a/x faculty. On average, participants attended 6.6 sessions (range 6–10). A general comparison of our attendees to overall faculty at NUFSM suggests we enrolled a higher percentage of women, Team Science, and Research Track faculty, fewer Clinician Educators, and a similar percentage of Investigator/Tenure Track faculty.

**Table 2.** Demographic characteristics of *N* = 46 participants who completed the mentoring training certificate program

	N	(%)
**Current rank**		
Instructor	1	(2%)
Assistant Professor	26	(57%)
Associate Professor	13	(28%)
Full Professor	6	(13%)
**Career track**		
Clinician Educator Track	20	(43%)
Investigator (Tenure Track)	8	(17%)
Research Track	9	(20%)
Team Science Track	4	(9%)
Health Systems Clinician	4	(9%)
Other	1	(2%)
**Years experience**		
0 years (no formal experience)	6	(13%)
1 - 5 years	23	(50%)
>5 - 10 years	9	(20%)
>10 - 15 years	5	(11%)
> 20 years	3	(7%)
**Current mentor**		
No	2	(4%)
Yes	43	(96%)
**Self-identified gender**		
Man (He, him)	16	(35%)
Woman (She, her)	29	(63%)
Prefer not to answer	1	(2%)
**Self-identified race**		
Alaska Native/Native American	0	(0%)
Asian	11	(24%)
Black/African American	0	(0%)
Native Hawaiian/Pacific Islander	0	(0%)
White	33	(72%)
Other race	1	(2%)
Prefer not to answer	2	(4%)
**Self-identified ethnicity**		
Hispanic or Latino/a/x	2	(4%)
Not Hispanic or Latino/a/x	43	(93%)
Prefer not to answer	1	(2%)

### Mentoring skills

For each of the 9 skills assessed, Table 3 shows mean Likert scores at baseline, at the end of the program, and their differences. At baseline, mentors ranked themselves as moderately skilled (average Likert values near 3) in *articulating needs*, *improving communication*, *providing feedback*, *coordinating with mentors*, *helping balance*, and *creating opportunities*. They rated themselves as slightly more skilled in *building rapport* (mean = 3.9) and slightly less skilled in *distinguishing mentoring plan/philosophy* and *considering biases* (mean 2.7 and 2.8, respectively).

**Table 3.** Differences in mentoring skills from baseline to end of program (*N* = 46)^1, 2^

	Baseline		End of Program					
Skill	Mean	(SE)	Mean	(SE)	Diff	(95% CI)		*p*-value
Articulating needs	3.13	(0.14)	3.89	(0.10)	0.76	(0.45,	1.07)	<0.05
Building rapport	3.92	(0.21)	4.38	(0.18)	0.46	(0.06,	0.86)	<0.05
Improving communication	3.13	(0.13)	4.09	(0.10)	0.96	(0.63,	1.28)	<0.05
Providing feedback	3.33	(0.12)	3.78	(0.10)	0.46	(0.18,	0.73)	<0.05
Coordinating with mentors	2.93	(0.15)	3.85	(0.10)	0.91	(0.57,	1.25)	<0.05
Helping balance	3.17	(0.13)	3.93	(0.12)	0.76	(0.42,	1.10)	<0.05
Distinguishing mentoring plan/philosophy	2.67	(0.26)	3.92	(0.15)	1.25	(0.58,	1.92)	<0.05
Creating opportunities	2.89	(0.21)	3.53	(0.21)	0.63	(0.07,	1.19)	<0.05
Considering biases	2.78	(0.14)	3.62	(0.10)	0.84	(0.60,	1.09)	<0.05

1 Mentoring skills were assessed on a scale of 1 to 5 for which 1 = Not at all skilled; 3 = Moderately skilled; and 5 = Extremely skilled.

2 Questions on building rapport, distinguishing between mentoring plan/philosophy, and creating opportunities were not initially asked at baseline, and are therefore available on 13, 12, and 19 participants, respectively.

Scores increased significantly at the end of the program for every skill. Increases ranged from about a half a point (*building rapport* 0.46 95% CI 0.06 – 0.86; *providing feedback* 0.46, 95% CI 0.18 – 0.73) to nearly a full point (*improving communication* 0.96, 95% CI 0.63– 1.28; *coordinating with mentors* 0.91 95% CI 0.57 – 1.25), to more than a point (*distinguishing mentoring plan/philosophy,* 1.25, 95% CI 0.58–1.92). After program completion, average values were 3.5 or higher for every mentoring skill, and 4 or higher for *building rapport* and *improving communication.*

### Frequency of specific mentoring behaviors

**Figure 1.**
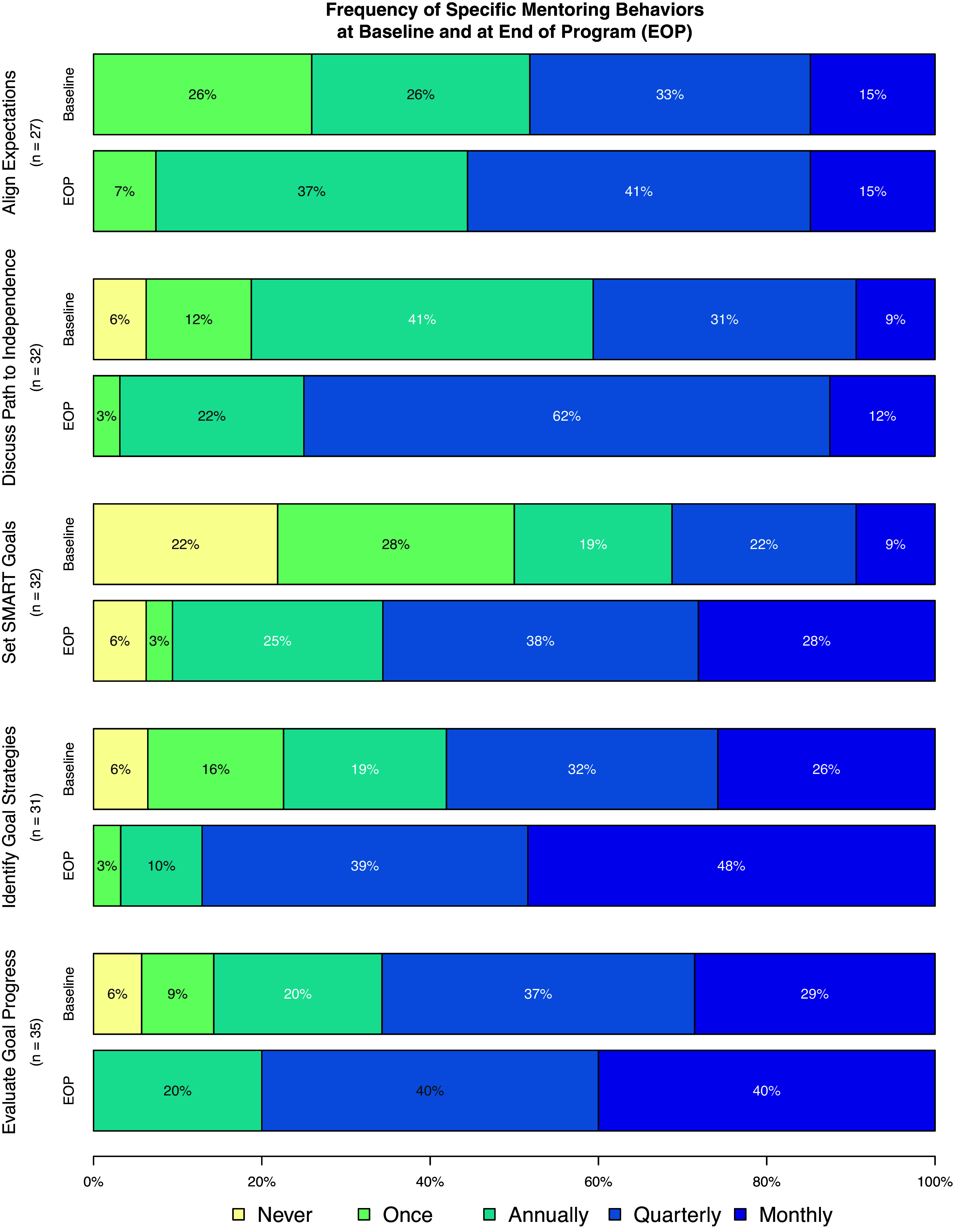
Frequency of specific mentoring behaviors at baseline and end of program (EOP) for participants in the mentoring training certificate program. Sample sizes vary because mentors could respond with “not applicable” and “other” a survey programming error resulted in 5 participants not being asked all items; and participants were not asked these questions if they were not actively mentoring at the time (*n* = 2).

Fig. 1 illustrates the frequency of types of interactions with mentees at baseline and at the end of the program. At baseline, all mentors who responded were *aligning expectations* at least once, whereas nearly a quarter of mentors had never worked with their mentees to *set SMART goals*. After completion of the program, frequency of mentors’ interactions increased significantly, regardless of the type of interaction. Mentors had increased odds of more frequent *aligning of expectations* (OR 2.8, 95% CI 1.2 – 6.7), *discussing a path to independence* (OR 4.1, 95% CI 1.6 – 10.4), *setting SMART goals* (OR 5.4, 95% CI 2.1 – 14.1), *identifying goal strategies* (OR 5.3, 95% CI 1.9 – 14.5) and *evaluating goal progress* (OR 3.6, 95% CI 1.3 – 9.4).

After program completion, mentors were also more likely to use Individual Development/Career Plans (IDPs) and meeting agendas. At baseline, 62.5% of mentors never or seldom used an IDP, 17.5% used an IDP about half the time, and 20% used an IDP most of the time (i.e., usually or always). In contrast, after program completion, 27.5% of mentors never or seldom used an IDP, 30% used one about half the time, and 42.5% used one most of the time (OR for increase in frequency 12.9, 95% CI 4.0 – 41.7). Similarly, requesting agendas from mentees was less common at baseline (61% seldom or never; 7% about half the time; 32% usually or always) than at program completion (19.5% seldom or never; 19.5% about half the time; 61% usually or always). Mentors had increased odds of using agendas more frequently after program completion (OR 13.1, 95% CI 4.0 – 43.4).

### Overall assessment

Mentors reported high satisfaction with the program. More than 80% said they were “very likely” to recommend the program to a colleague, and all but 2 reported they were “likely” or “very likely” to recommend the program (95%, *n* = 42/44 respondents). Similarly, 96% of respondents reported that the series was “extremely valuable” (72%) or “valuable” (24%) to them as a mentor.

### Qualitative responses: mentoring “Pearls,” feedback, and suggestions for future programming

Mentoring pearls identified included the concept of having a “mentoring philosophy,” the need to articulate goals and align expectations, and the importance of recognizing cultural differences and one’s own biases. Others highlighted the benefits of setting agendas and creating and revising career plans with mentees. On participant simply stated their pearl was “learning what I didn’t know I didn’t know.”

Overall, participant feedback regarding the program was highly positive: “Great Work!” “Phenomenal Program!” “It’s been a great experience to participate in these mentoring series.” Additional feedback identified an appreciation of the interactivity provided during the workshops and enjoyment of the peer mentoring aspect experienced when working in small groups, coupled with requests for increased opportunities to role play during workshops.

### Modifications of MTCP

Many attendees appreciated the flexibility and convenience that virtual attendance provided them. As we intend the workshop series to be a catalyst in creating a community of practice of mentors across NUFSM, we plan on presenting workshops in person for the foreseeable future. Although some virtual sessions, particularly in the fourth year of our program, had increased attendance, attendance at our in-person sessions has increased over time. Our average in-person attendance across workshops in the fourth year of our program (30 attendees) was greater than average attendance in any preceding year (range 21–24 attendees).

Based on attendee feedback, we rotate the days of the week that the mentoring workshops are held annually, recognizing that many attendees have clinical or clinical supervision responsibilities that make it challenging to reschedule. We continue to “crowd source,” asking faculty to suggest mentoring topics of interest.

## Discussion

The NUCATS Developing and Enhancing Mentoring Relationships Mentor Workshop Series and MTCP was designed to provide foundational training for faculty mentors and offer ongoing opportunities for faculty to continue to advance their mentoring practices. In the first four years of the program, we succeeded in engaging 345 unique faculty across ranks and tracks. Similar to other mentorship training programs [4,6], our MTCP participants reported significant increases in multiple mentoring skills. We observed the largest gains in reports of mentors *distinguishing between mentoring plan/philosophy, employing strategies to improve communication with mentees,* and in *coordinating effectively with my mentee’s other mentors.* These skills are particularly crucial as relevant to the National Center for Advancing Translational Sciences (NCATS) objective 4.1 to “support and promote **team** science” [15]. Communicating effectively with other faculty, in this case both mentees and co-mentors, is a crucial skill needed for collaborative efforts to produce translational research.

We demonstrated a reported increase in the frequency of recommended mentoring behaviors endorsed throughout our mentoring series, including *aligning expectations*, *discussing mentee paths to independence*, and *setting and evaluating goals*. Participants reported large increases in the use of IDP/CAPs and agendas in their mentoring relationships, with some identifying their use of these tools as a “mentoring pearl” gained from the program.

Similarities between the MTCP and mentor training programs at other universities include providing workshops that focus on evidence-based mentoring competencies and skills, a similar time commitment, and using case studies and interactive breakout sessions to engage mentors’ reflection [3–6]. In contrast to other programs, we change our content annually, save for three required sessions presented yearly. Providing a variety of monthly workshops was in response to previously trained faculty seeking opportunities for additional training. Further, we intentionally include faculty who mentor across research, clinical, and educational contexts. As we enroll more faculty, we will have the opportunity to explore differences between those attending the workshops who do or do not complete the MTCP.

In response to participant suggestions, we created workshops on navigating conflict and challenges in mentoring relationships, team-based mentoring and mentoring in groups, giving meaningful feedback, mentoring in mid-career, and being an ally and a sponsor. In addition, given the high level of interest in the MTCP among early-career faculty, we recently launched a “Mentee Development Series,” of three workshops designed to prepare early career faculty to be effective mentees.

### Limitations

Our findings are limited by the use of self-reported measures, recognizing that participants may be unable to accurately assess their own skills. An additional limitation is the self-selection of participants engaged in the MTCP. It is possible that mentors would expect benefit from participation in mentor training workshops and thus may rate their skills higher after participation. However, unlike other training programs where baseline and follow-up measures are assessed within 3 months or less [4,6], the shortest time between completion of baseline and annual surveys was 7 months. We do not have data to demonstrate that these self-reported gains in mentoring skills will be retained over time. We are unable to assess mentees’ perceptions of improvement in mentors’ skills as we only surveyed mentors. However, previous research assessing mentors’ skills within mentee–mentor pairs suggests a weak correlation among such paired assessments and notably demonstrates that mentees consistently rate mentors’ competency in mentoring skills higher than the mentors’ self-assessment [11,16]. Although mentors’ self-assessed skills improved, we did not measure how these changes might also improve mentee outcomes. Finally, because we customized items from the MCA, the modified instrument could not be evaluated for performance, and we were unable to directly compare the effects of our curriculum to others.

## Conclusion

In the first four years of a Mentor Training Certificate Program, we demonstrated a self-reported increase in mentoring skills and a parallel increase in participants’ frequency of recommended mentoring behaviors. Annual provision of a core set of workshops, coupled with a variety of elective workshops,

was well received by faculty, who had flexibility in attending sessions of their choosing. Our program is responsive to expectations from federal funders for ensuring mentors on training grants receive formal training [9]; the Northwestern Office of the Provost and Graduate School recognize completion of the program as meeting the new NIH mentor training requirements. To meet the expanded need for mentor training, our institution used our successful MTCP as a model to launch additional training offerings, which were created in collaboration with the Office of the Provost, the Center for Leadership, the Graduate School, and NUCATS. The NUCATS mentor workshop series will further be of benefit as funders expect faculty to engage in “refresher training” over time. Our program, offering both repeating and new workshop options, will provide an opportunity for mentors seeking either initial or continuing training.

## Supporting information

Cameron et al. supplementary material Cameron et al. supplementary material

## Supplementary material

The supplementary material for this article can be found at https://doi.org/10.1017/cts.2025.71.
