# Supplementary material for: Development and assessment of a Mentor Training Workshop Series and Certificate Program
Source: J Clin Transl Sci. 2025 Apr 16;9(1):e98. doi: 10.1017/cts.2025.71 (PMC12089858; doi:10.1017/cts.2025.71)
Supplement: Cameron et al. supplementary material [file S2059866125000718sup001.docx]

**SUPPLEMENTARY APPENDIX. Measurement of Mentoring Skills and Behaviors**

**Mentoring Skills^1^**

Please rate how skilled you feel you are in each of the following areas:

Please only choose “not applicable” (NA) when a skill cannot be applied to any of your interactions with mentees/mentors.

*1 = Not at all skilled, 3= moderately skilled, 5 = extremely skilled*

Engaging my mentee(s) in articulating their mentoring needs.

Building rapport with my mentee(s).

**Employing strategies to improve communication with** *my* **mentee(s).**

**Providing constructive feedback** *to my mentee(s) (either positive or negative)*

**Coordinating effectively with** *my* **mentees’ other mentors.**

**Helping** *my* **mentee(s) balance work with personal life.**

Distinguishing between a mentoring plan and a mentoring philosophy.

Intentionally creating opportunities for my mentee(s) to bring up issues of race/ethnicity and other social identities.

*Considering the conscious and unconscious biases I bring to the mentoring relationship.*

^1^If **bold**, then item is verbatim from Mentoring Competency Assessment [cite]. If in *italics*, then item was adapted from Mentoring Competency Assessment. Regular text items were added to address known areas addressed in our mentor training.

**Frequency of Engaging in Mentoring Behaviors^2^**

Thinking in general of your mentoring relationships, how often do you…

…formally (verbally or in writing) align your expectations/revise expectations with your mentee(s)?

…specifically discuss your mentee(s)’ path to professional independence?

…work with your mentee(s) in setting SMART (Specific, Measurable, Achievable, Relevant, Time-Bound) goals?

…work with your mentee(s) in identifying specific strategies to reach their identified goals?

…work with your mentee(s) in evaluating their progress toward their identified goals?

^2^Items were adapted from the Mentoring Competency Assessment [11] and revised to assess frequency of each identified behavior. Answer options included “never,” “seldom,” “about half the time,” “usually,” “always,” and “other.”

***Mentoring behaviors***

*How often do you (Never, Seldom, about half the time, usually, always) [other]*

*…use a mentoring compact or individual/Career Development plan with your mentees*

*…request an agenda from your mentee(s) for scheduled meetings?*

**Example Workshop Timeline: Establishing and Aligning Expectations in Mentoring Relationships**

| **Time** | **Activity** |
| --- | --- |
| 10 min | Welcome, Schedule, Reminders, Overview of Mentor Training at Northwestern/Mentor Training Certificate Program |
| 5 min | Agenda, Northwestern's 4 Pillars, Learning Objectives |
| 5 min | Mentoring for What/Mentoring Roles and Mentoring Needs  What is needed? Who can help? Are you the right person? |
| 15 min | Establishing and Aligning Expectations: Narrative example, presentation and discussion of Expectancy Violation Theory  **Audience Response System Prompt/Discussion:**  What needs to be aligned in mentoring relationships/Candidates for alignment?  Why align expectations? |
| 25 min | **Case Studies**  **Breakout Groups** (10 min)  **Large Group Discussion/Report Back** (15 min) |
| 15 min | Alignment Tools: Presentation and **Discussion** of use of Checklists, Agendas, Career Advancement Plan, Laboratory Expectations/Guidelines, Mentoring Compacts |
| 5 min | Aligning (and Realigning) Expectations: Best Practices |
| 5 min | Next Steps and Action Plan  **Audience Response System Prompt/Discussion:**  What behavior change will you enact in the next 3 - 6 months to improve alignment in your mentoring relationships? |
| 5 min | Wrap Up/**Questions** |

**Example Case Study: Establishing and Aligning Expectations**

The Mentor who loves mentoring

You are a faculty member at Feinberg School of Medicine who has been very active in mentoring throughout your career. You thoroughly enjoy the opportunity to mentor others, and many people come to you for mentoring. You are currently serving as a primary mentor or co-primary mentor to five early career faculty members, are a very active co-mentor to an additional two early career faculty and serve as primary mentor for three graduate students. ​

You take your role as mentor seriously, meeting with each of your mentees at least twice a month, and you always give written feedback when asked (i.e., when reviewing abstracts, manuscripts, case reports, proposed course assignments, or curricula). Recently you have had some commitments that have filled up your schedule and you have not been able to be as quick with turnaround of comments– you took three weeks longer than originally planned to provide your faculty mentee feedback on a manuscript. ​

Your mentee just appeared at your door for your bi-monthly meeting, and you told her that another student just asked you to serve as a primary mentor, and you were very excited to have been asked. Your mentee paused, then said, “You already have a lot of mentees, and, quite frankly, I am worried that you are considering taking on even more, because that means you will have even less time for me moving forward.”

What are the main themes raised in this case study?

How might you respond as the faculty mentor?

What are your perceptions of the faculty mentee at this point?

Other general comments?
